# Plant sphingolipids: Their importance in cellular organization and adaption^☆^

**DOI:** 10.1016/j.bbalip.2016.04.003

**Published:** 2016-09

**Authors:** Louise V. Michaelson, Johnathan A. Napier, Diana Molino, Jean-Denis Faure

**Affiliations:** a Biological Chemistry and Crop Protection, Rothamsted Research, Harpenden AL5 2JQ, UK; b Ecole Normale Supérieure-PSL Research University, Département de Chimie, Sorbonne Universités - UPMC Univ Paris 06, CNRS UMR 8640 PASTEUR, Paris, France; c INRA, Institut Jean-Pierre Bourgin, UMR 1318, ERL CNRS3559, Saclay Plant Sciences, Versailles, France; d Agro Paris Tech, Institut Jean-Pierre Bourgin, UMR 1318, ERL CNRS3559, Saclay Plant Sciences, Versailles, France

**Keywords:** Fatty acid, Long chain base, Glycans, Membrane, Phosphorylation

## Abstract

Sphingolipids and their phosphorylated derivatives are ubiquitous bio-active components of cells. They are structural elements in the lipid bilayer and contribute to the dynamic nature of the membrane. They have been implicated in many cellular processes in yeast and animal cells, including aspects of signaling, apoptosis, and senescence. Although sphingolipids have a better defined role in animal systems, they have been shown to be central to many essential processes in plants including but not limited to, pollen development, signal transduction and in the response to biotic and abiotic stress. A fuller understanding of the roles of sphingolipids within plants has been facilitated by classical biochemical studies and the identification of mutants of model species. Recently the development of powerful mass spectrometry techniques hailed the advent of the emerging field of lipidomics enabling more accurate sphingolipid detection and quantitation. This review will consider plant sphingolipid biosynthesis and function in the context of these new developments. This article is part of a Special Issue entitled: Plant Lipid Biology edited by Kent D. Chapman and Ivo Feussner.

## Highlights

• Up-to-date review of our current understanding on plant sphingolipid biosynthesis
• In depth consideration of plant-specific aspects of sphingolipid metabolism
• Updates on the biosynthesis of GIPCs in plants
• The sometimes overlooked role of VLCFA biosynthesis in sphingolipid biogenesis

## 1 Introduction

Sphingolipids and their intermediates are an incredibly diverse group of molecules with a vast array of physical properties. The wide range of sphingolipid structures facilitates their function in a variety of cellular processes. The different classes and modifications offer a multitude of differing solubility, charge, shape, and size. This enables sphingolipids to be involved in activities as diverse as structural integrity of the membranes [63] and in membrane domain formation [6], [36] and as metabolites mediating cellular process such as programmed cell death and abscisic acid dependent signal transduction [17], [70] and in the response of plants to hypoxia [72], [73] and to pathogen attack [60]. Sphingolipids form a significant proportion of the lipids present in higher plants, with some studies suggesting that they constitute up to 10% of plant lipids [19]. Novel plant lipid structures are still being discovered and over two hundred have been identified in various different species to date [11], [45].

The physician and biochemist J.L.W. Thudichum coined the term sphingo in 1884 because the impenetrable nature of this lipid reminded him of the riddle of the sphinx. The term sphingolipid was introduced by Herbert Carter, a pioneer in lipid research, and colleagues in 1947 when he elucidated the structure of sphingosine. Historically sphingolipids were studied in the brain where they are an abundant lipid class. Until relatively recently sphingolipids have not been studied extensively in plants. Of the major lipid classes, sphingolipids were often overlooked perhaps due to having many species which do not partition with neutral and glycolipids in classical lipid extraction techniques. Over the last 10 years more comprehensive extraction techniques have been developed which when coupled with technological advances in mass spectrometry and chromatographic techniques have enabled more accurate sphingolipid quantification and the discovery of novel structures.

### 1.1 Sphingolipid structure

**Fig. 1:**
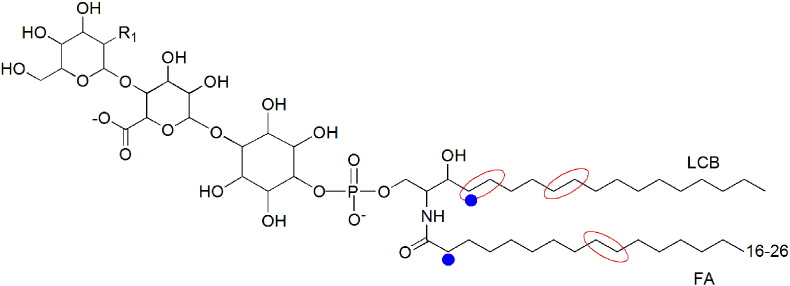
Diagram of a plant sphingolipid.

Sphingolipids are structurally characterized by a sphingoid base acyl chain amide linked to a fatty acid (FA). This structure is termed ceramide (Cer) and can be further modified by the addition of glycosyl residues and other polar phosphate-containing head groups. The array of physical properties that sphingolipids exhibit are enabled by the many different structures which are present in plants. The long-chain base (LCB) component of the sphingolipid can vary in chain length, usually between 16 and 20 carbons and can undergo hydroxylation and/or desaturation. The amide-linked fatty acid or very long-chain fatty acid (VLCFA) can also undergo modifications, varying in length from 16 to 30 carbons and can be hydroxylated at the C2 position and desaturated at the ω-9 position leading to a multiplicity of sphingolipid isoforms (see Fig. 1). Hydroxylation of FA and LCB moieties are the hallmark of sphingolipids. These modifications can profoundly affect the subsequent biological activity. The main aspects of sphingolipid biosynthesis and metabolism in animals, yeast and plants are largely conserved and much of the knowledge gathered from research in both the animal kingdom and yeast has benefitted plant sphingolipid biology. One of the key differences between plant sphingolipids and sphingolipids from other eukaryotes is that they exhibit a much larger structural variability and the rationale for this diversity is still poorly understood.

Complex sphingolipids in plants are formed by a ceramide back bone comprised of a long chain base (LCB) esterified to a long chain fatty acid (long chain FA or VLCFA). As illustrated here the acyl chains are subject to a number of potential modifications, the red lines show the site of potential desaturations and the blue dots the hydroxylations. The LCB and the ceramide can exist in the phosphorylated or non-phosphorylated form. The ceramide can be glucosylated or as shown here incorporated into the complex sphingolipid glycosyl inositol phosphoryl ceramide (GIPC) where it is linked to a inositol–glucuronic acid unit via a phosphodiester bond. R_1_ can be a hydroxyl, amine or an N-acetylamine group. Additional saccharides can be linked to inositol.

Plant sphingolipids can be divided into four main classes; these are ceramides, glycosylceramides (GlcCers), glycosyl inositolphosphoceramides (GIPCs), and free long-chain bases (LCBs). The glycosylated inositolphosphorylceramides (GIPCs) are considered to be the predominant sphingolipids in plant tissue [46] (Fig. 1). This is in contrast to the situation in animals, where the main classes are sphingomyelin (a class of phosphorylceramide not found in higher plants; [64] and the neutral and acidic glycolipids, and in yeast where inositolphosphorylceramides (IPCs) and their mannosylated derivatives predominate [37]. In plants, the amounts of the sphingolipid classes tend to vary in a species and a tissue-dependent manner [27], [46]. In *Arabidopsis* leaves GIPCs are the predominant class, with GlcCers present at approximately half the GIPC level. The remaining sphingolipids are present mainly as ceramides, with free LCBs and phosphorylated LCBs representing minor components [45]. The different tissues in the plant show different sphingolipid composition. Pollen fractions are highly enriched in glucosylceramides relative to levels previously reported in leaves [42] and seeds have been shown to have differing sphingolipid profiles [65]. Plant cell cultures identified some complex GIPCs that have yet to be found in leaf tissue [10], [55]. These observations from different tissue types raise the question of the functional significance of alternative structures and compositions but as yet no comprehensive explanation has been proposed.

The LCB and the fatty acid components of sphingolipids are subject to compositional variation depending on the organism. LCBs in plants are predominantly C18 amino alcohols and they are largely comprised of 4-hydroxysphinganine (t18:0), commonly known as phytosphingosine, and its desaturated form 4-hydroxy-8-sphingenine (t18:1^8^). The latter LCB is found almost exclusively in the plant kingdom, whereas t18:0 is found in some animal species, despite the “phyto” appellation. Other LCBs present in plants include sphinganine also known as dihydrosphingosine (d18:0) and its desaturated forms 8-sphingenine (d18:1^8^), 4-sphingenine also known as sphingosine (d18:1^4^) and 4,8-sphingadienine (d18:2^4,8^). The double bond at the Δ^8^ position can be present in either the cis (Z) or the trans (E) configuration and the ratios of these isomers vary according to the species. The double bond at the Δ^4^ position is present in the trans (E) configuration. A few rarer LCBs have also been reported in plants [32] and in algae [50], but it is the nine LCBs described here that represent the main pool of LCBs in plants. The ratio of cis (Z) to trans (E) isomers of Δ^8^-unsaturated LCBs can change depending on the sphingolipid it is a component of, and this in turn, may influence the subcellular location of the sphingolipid [63]. It has been suggested that the ratio of the isomers of Δ^8^-unsaturated LCBs is correlated with the chilling tolerance in plants [33]. This correlates with the observation that the *Arabidopsis* double *sld1 sld2* mutants, which showed no detectable LCB Δ^8^ unsaturation, were unable to tolerate prolonged exposure to low temperature which was in contrast to wild type plants [14].

The Δ^8^-unsaturated LCBs are only widely found in the plant kingdom; they are absent from animals and the yeast *Saccharomyces cerevisiae* (which represents the best characterized organism in terms of sphingolipid biosynthesis), though some fungi such as *Pichia pastoris*, *Candida albicans* and some marine algae have been shown to contain Δ^8^-unsaturated LCBs [66]. The predominant LCB of many animal sphingolipids is sphingosine (d18:1^4^) and this is usually only found as a minority component of plant sphingolipids [46].

The fatty acyl component of the ceramide, and therefore the sphingolipid, is generally α-hydroxylated (C-2 position) and tends to vary in chain length from 16 to 30 carbons [29], [43]. Chains may be desaturated at the ω-9 position, and there is evidence to suggest that this modification of the fatty acid predominates in the glucosylceramides of cold adapted cereal plants [28]. The significance of this chemical diversity, however, remains to be fully explored. There are two main types of complex plant sphingolipids, as mentioned above. The glycosylceramides carry between 1 and 4 glycosyl residues attached to C1 of the N-acyl hydroxyl group of the LCB of the ceramide. The GIPCs carry inositol-1-phosphate linked as a phosphodiester to the primary carbon of the ceramide. This can be extended by oligosaccharide chains and these are usually linked at position 2 and/or position 6 of the inositol moiety.

### 1.2 LCB synthesis

**Fig. 2:**
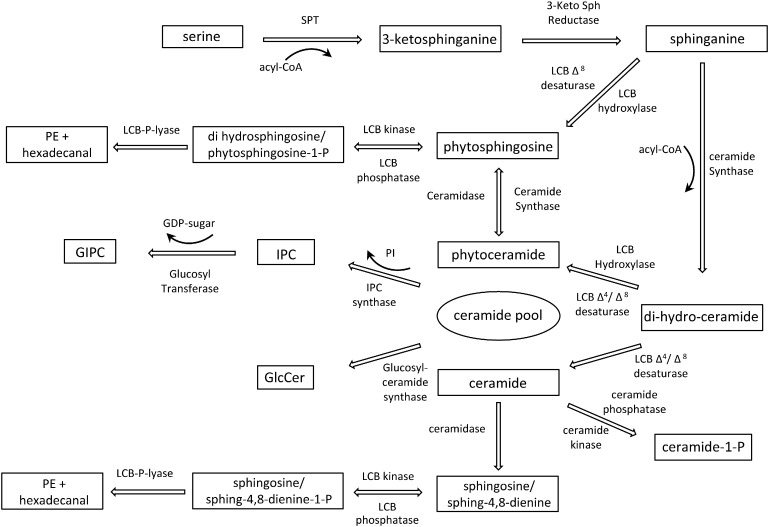
Schematic representation of sphingolipid biosynthesis of in plants.

The first step of the biosynthesis of sphingolipids is the generation of the sphingoid backbone by the condensation of serine and palmitoyl-CoA (Fig. 2). This is catalyzed by serine palmitoyl transferase (SPT) and generates the long chain base 3-ketosphinganine which is reduced by the ketosphinganine reductase (KSR) to form sphinganine. Inactivation of *SPT* resulted in lethality in both animals and plants [13], [26] demonstrating the importance of sphingolipids and LCBs for cell viability. *Arabidopsis* mutants lacking SPT, displayed embryo and male gametophyte lethality [13], [18]. Even a reduction of SPT activity has an impact on plant growth since a 20% reduction was sufficient to impair cell expansion in *Arabidopsis* [13]. SPT functions as a dimer and in *Arabidopsis*, mutations in the small subunits of SPT (ssSPT) also constrain sphingolipid biosynthesis and impair male gametophyte viability [34]. As the first committed step in sphingolipid synthesis, the regulation of SPT is essential. ORM proteins were found to be negative regulators of SPT in yeast [7]. In rice, RNAi against ORM-like genes also led to abnormal pollen development confirming the importance of sphingolipids for male fertility [16].

The 3-ketosphinganine reductase catalyzes the second step of LCB synthesis and the 3-ketosphinganine is reduced to sphinganine (d18:0) the simplest LCB found in plants. Two genes in *Arabidopsis* termed KSR1 and 2 encode the 3-ketosphinganine reductase [3], [19]. The LCB can be incorporated into ceramides. The LCB modification is present either as a hydroxylation which likely occurs prior to ceramide synthesis or desaturation which likely occurs on a more complex sphingolipid.

The inferred biosynthetic pathway for the synthesis of complex sphingolipids. The modifications of the Long Chain Base (hydroxylation, desaturation) are shown acting on the di-hydro-ceramide with the LCB Hydroxylase additionally showing potential activity on sphinganine. PI – phosphatidyl Inositol, IPC – inositol phosphorylceramide, GIPC – glycosyl inositol phosphorylceramide, GlcCer – Glycosyl ceramide.

### 1.3 LCB modifications

The LCB moiety contains 18 carbon atoms chain that can be further hydroxylated and desaturated. A total extract of LCBs from *Arabidopsis* reveals a majority of di- and tri-hydroxylated LCBs (d18 or t18). Di-hydroxylation is associated with the presence of hydroxyl groups on C1 and C3, while the third hydroxylation which predominates in plant LCBs is on C4. In *Arabidopsis*, redundant genes, *SBH1* and *SBH2* (for *S**PHINGOID* *B**ASE* *H**YDROXYLASE 1 and 2*), encode the C4-hydroxylase. Double mutants and RNAi suppression lines demonstrated the importance of this hydroxyl group [15]. The absence of C4 hydroxylation and thus trihydroxy-LCB resulted in cell division and cell expansion defects that could not be compensated by elevated levels of di-hydroxy-LCBs. The hydroxylation level in LCBs is critical in the sphingolipid biosynthetic pathway since di-hydroxy-LCBs are preferentially incorporated in shorter fatty acyl chain sphingolipids. These observations suggested that trihydroxy-LCB and dihydroxy-LCB would be preferentially channeled in VLCFA- and LCFA-sphingolipids respectively.

The LCB desaturation occurs at either C4 or C8. LCBs cannot undergo both hydroxylation at C4 and desaturation at the Δ4 position making these modifications mutually exclusive. Plants must exert some measure of control or spatial separation to enable the correct ratio of LCBs to be synthesized or incorporated into specific lipids at specific locations. The Δ^4^-desaturase in *Arabidopsis* (DES4) is expressed in specific tissues, mainly pollen and at low levels in flowers [51]. Interestingly, the *des4* mutant showed specific sphingolipid alterations with a reduction of long chain sphingolipids but not those with very long acyl chains indicating that like C4-hydroxylation, Δ4-desaturation channels LCB toward sphingolipids containing a C16 acyl chain. A C4 modification of the LCB could thus provide structural information for a C16-selective ceramide synthase, though it is likely that a range of other factors (including substrate availability, cell type and developmental stage) also contribute the final composition of GlcCers in plants. The Δ^8^-desaturation is the principal desaturation of LCBs in plants. The distribution of LCB and their *cis*/*trans* isomers is however highly variable between plant species. The two isomers t18:1Δ^8E^ and t18:1Δ^8Z^ represent respectively 20 and 3.4% of *Arabidopsis* GlcCer a ratio that is inverted in maize [58]. A survey from 21 different plant species showed that Δ^8^-desaturation of dihydrosphingosine seems to be widely distributed in plants but ∆^4^-desaturation of dihydrosphingosine occurs in some species in particular in the lower (non-seed) vascular plants and in the Poales [30].

### 1.4 Very long chain fatty acid synthesis

The fatty acyl component required for the ceramide is usually 16–26 carbons long and they are generated in the ER by an elongation complex of four core enzymes, which sequentially add two carbon units to a growing acyl chain. Briefly the VLCFAs are generated by the condensation of malonyl CoA with an acyl-CoA chain followed by the reduction of the 3-ketoacyl-CoA product. The 3-hydroxyacyl-CoA intermediate is then dehydrated resulting in an enoyl-CoA product which is reduced to give the two carbon elongated acyl-CoA chain, reviewed in [25].

In *Arabidopsis*, variable numbers of genes are observed to encode the activities for the elongase complex. To date it has been found that the 3-keto acyl-CoA synthase (KCS) is encoded by 21 genes [31]. Two genes have been cloned for 3-keto acyl-CoA reductase (KCR1 and 2) [2], 3-hydroxy acyl-CoA dehydratase (HCD/PASTICCINO2 and PTPLA) [1], and one for enoyl-CoA reductase (ECR/CER10) [74] but it is likely that there are other genes yet to be characterized that have involvement in elongation.

The components of the elongase have been elucidated both by the use of mutagenized populations and by reverse genetic techniques based on plant homologs to characterize components in other model species. Much of this work has underlined both the importance of VLCFAs to plant viability and survival and their critical importance in sphingolipid biosynthesis and function. For example, a weak *pasticcino2* allele, defective in the 3rd enzyme of the elongase complex, presents a reduction of VLCFA in sphingolipids and in other lipid classes (waxes, triacylglycerides and phosphoglycerolipids). While the organ fusion defect is thought to be caused by the reduction of VLCFA in waxes [1], the defect of division and differentiation observed in the mutant [1], [4] is most probably due to the shorter fatty acid chain length in membranes. The *ecr* mutant in *Arabidopsis*, which is impaired in the last enzyme of the elongase complex sequence, CER10, shows abnormal cell expansion linked to aberrant endocytic membrane trafficking [74]. The authors proposed that changes in the length of sphingolipid FAs were likely responsible for the membrane defect. It is interesting to note that VLCFA depletion, does not affect the different classes of sphingolipids equally. In the elongase defective *pas2* mutant, VLCFA content was not modified in ceramides or hydroxyceramides and moderately reduced in GIPC [1]. The GlcCer pool showed a strong depletion of VLCFA suggesting that this class of sphingolipid is either more unstable with higher turnover or its homeostasis is less tightly controlled providing an adjustable VLCFA pool.

### 1.5 Ceramide synthesis and degradation

Ceramide is synthesized by a condensation reaction of the LCB with the fatty acyl chain catalyzed by ceramide synthase (Fig. 2). The α-hydroxylation of the acyl chain produces hydroxy-ceramide or hCer [43], [45]. Ceramide and hydroxyl-ceramide represent only 1–2% of total sphingolipids since they serve mostly as biosynthesis intermediates or signaling molecules. The hydroxylation of sphingolipids is important for the specific association with sterols in membrane microdomains [6]. In *Arabidopsis* three ceramide synthases were identified in the genome and were named *LOH1–3*. LOH2 is specific to C16 acyl chains with a preference for dihydroxy LCB while LOH1 and 3 showed specificity on 20 to 26 carbon acyl chains in association with a trihydroxylated LCB. The *LOH1 LOH3* double mutant was depleted in very long acyl chain sphingolipids and non-viable in comparison to *LOH2* mutants lacking long acyl chain sphingolipids thus demonstrating the importance of acyl chain length [47]. Overexpression of *LOH1* and *LOH3* in *Arabidopsis* produced larger plants with an increase in cell division while *LOH2* over expression impaired growth and led to dwarf plants [40], [41], [42]. The LOH2 impact on plant growth is thought to be due to the accumulation of C16 dihydroxy sphingolipids that were able to induce programmed cell death. It was thought that the VLCFA-sphingolipid accumulation which led to enhanced growth could be explained at least partially by the importance of these lipids in membrane structure and dynamics. Sphingolipids containing VLCFA were found to be necessary for sorting specific membrane auxin carriers from the trans-Golgi network toward the plasma membrane in vivo [47] but also for vesicle fusion during exocytosis and cytokinesis [53].

The toxic and carcinogenic mycotoxin isolated from *Fu**sarium* *mon**iliforme* [23] is able to directly inhibit the enzymatic activity of ceramide synthase in vitro, and the potency of the inhibition was correlated with the accumulation of free LCBs and acyl-chain substrates [49]. The mycotoxin fumonisin B1 (FB1) was found to inhibit the different ceramide synthases differentially with a strong preference toward LOH1 and 3 ceramide synthases [47] and more specifically inhibiting LOH1 [40], [41], [42]. FB1 is a versatile tool in the analysis of sphingolipid metabolism and the role of sphingolipids containing VLCFAs in particular [53].

Ceramide homeostasis is maintained via the activity of ceramidases that are grouped in three classes, alkaline, neutral and acidic ceramidases, according to their optimum pH. An ER localized neutral ceramidase was first cloned in rice (OsCer) and its activity characterized by the complementation of yeast ceramidase double mutants ∆* ypc1* ∆* ydc1* [75]. Recently, it was found that the loss of function of the neutral ceramidase AtNCER1 in *Arabidopsis* enhanced plant sensitivity to oxidative stress [38]. Conversely, overexpression of *AtNCER1* protected the seedlings against methyl viologen treatments. Interestingly, AtNCER1 showed a greater specificity toward hCer than Cer since only the former was accumulated in *atncer1* mutant [38]. The functional analysis of a putative alkaline ceramidase in *Arabidopsis* showed an opposite picture with a stronger specificity toward Cer than hCer [71]. Reduced AtNCER1 activity led to high Cer levels and an increased sensitivity to salt stress but also to *Pseudomonas syringae* infection. It is clear now that Cer and hCer levels are important for the responses toward abiotic or biotic stresses in plants but further studies are needed to understand if these two sphingolipids define a rheostat tuning the plant for specific environmental changes.

### 1.6 Sphingolipid glycosylation and membrane function

Complex sphingolipids are synthetized from ceramides by the addition of simple or multiple sugars on ceramide at the C1 position. In plants the most abundant sphingolipids are glycosylceramide (GlcCer) and inositolphosphoryl-ceramide (GIPC). In Arabidopsis, glucosylceramide synthase (GCS) is encoded by a single essential gene, while GIPCs are synthetized by at least three functional IPC-synthases [52] and several Glycosyl transferase or Glucuronyl transferases [59] (Fig. 2). Plants lacking functional GCS could not develop beyond seedling stage but heterozygous plants carrying a null *gcs* allele had defective pollen transmission and also showed a reduced differentiation defect in regenerating callus tissue [56]. These phenotypes are reminiscent of *pasticcino* phenotype which is impaired in fatty acid elongation and therefore in sphingolipids containing VLCFA. They exhibit defective differentiation and organogenesis combined with ectopic cell proliferation [22], [24]. These developmental defects could directly be related to defective protein trafficking since GlcCer was found to be essential for Golgi morphology and protein secretion ([48], [56].

IPCs are synthesized via the head group transfer of phosphatidylinositol onto a ceramide catalyzed by IPC synthase. The first IPC synthase identified was ERH1, found in a screen for enhanced hypersensitive response mutants during pathogen infection [68]. The mutant *erh1* did not display any obvious change in GIPC levels, probably because of redundancy with two other predicted IPC synthases. However it accumulated substrate ceramides and this was the likely cause of the increased cell death responses in the mutant. Further glycan ornamentation of GIPC was elucidated with the identification of IPUT1 as inositol phosphorylceramide glucuronosyltransferase1 that transfers a glucuronic acid (GlcA) on GIPC [59]. The absence of even a single glycan like GlcA on GIPC has impact since it prevented pollen transmission. GIPC are characterized by a very important variability in their glycosylation pattern. A survey of 23 plant species identified at least 21 different patterns with variation in number, type and order of glycan substitutions [11]. Interestingly, in at least three plant species complexity of GIPC glycosylation increased when cells were cultured in vitro suggesting that GIPC could be involved in cell proliferation and differentiation.

GIPC and GlcCer can be distinguished by their head groups as well as by their fatty acid and LCBs. The LCB and fatty acyl chain channeling observed for ceramide and hCer could be observed for GlcCer and GIPC. In *Arabidopsis* seedlings, trihydroxy LCBs (mostly t18:1) are predominant in both GIPCs and GlcCers. GIPC are characterized by the presence of t18:0 largely with VLCFA while GlcCer shows the presence of dihydroxy LCB (d18:1 ∆^8^) in association with LCFA (C16) [45]. Pollen exhibits a sphingolipid profile with a strong enrichment in GlcCers specifically with C16/d18:2 that results in the pollen phenotype observed in *gcs* mutant [40], [41], [42], [56].

Recently the use of sensitive mass spectrometry has begun to identify and characterize the variety of head groups in the GIPC pool in various plants. The primary GIPC headgroup identified to date in *Arabidopsis* leaves contains a single hexose with hydroxylation bound to a hexuronic acid linked to IPC [9], [10], [11]. Other plants and cultures of *Arabidopsis* and tobacco contain additional GIPC structures which contain further hexoses and pentoses. These have been separated into six groups according to numbers and composition of the head group sugars [11] and can contain up to seven sugar residues bound to IPC. GONST1 is a Golgi localized GDP-sugar transporter that specifically supplies GDP-Mannose to the Golgi lumen for GIPC synthesis. Plants disrupted in GONST1 have a dwarfed phenotype and a constitutive hypersensitive response with elevated salicylic acid levels. This suggests a role for GIPC sugar decorations in sphingolipid function and potentially plant defense signaling [55]. This work showed that the head groups of the GIPCs are not only diverse but also have specific functions within the plant. The GIPCs are a much bigger and more complex lipid pool that previously thought though the functional significance of the many different GIPC sugar structures and numbers is currently unknown, mechanisms must exist to regulate this pool and control their distribution across different cell types.

Subcellular purification and analysis of cellular membranes showed variable lipid composition [54], [69]. While phospholipids are ubiquitous in the different membranes, sphingolipids and sterols showed levels ranging from very low or absent (such as in the ER membranes) to 10% of total lipid [43]. Recent reevaluation of membrane composition in BY2 cells and tobacco leaves showed that GIPC could account for 40% of total plasma membrane lipids [12]. The enrichment of sphingolipid in the plasma membrane is correlated with their important role in the formation of membrane microdomains [6], [36]. These microdomains are no larger than 35 nm, are highly resistant to detergent and showed a strong enrichment in highly glycosylated GIPC containing VLCFAs [12]. The very long acyl length has a likely role in lipid interdigitation between the two membrane monolayers and consequently is critical in microdomain cohesion [12].

In addition to the plasma membrane, sphingolipids are enriched in late endosomes and the plant tonoplast, where they represent 10–20% of the total membrane lipids [54], [69]. As with animal cells, a specific gradient of sphingolipid is observed along the secretory pathway with the highest accumulation at the plasma membrane. This gradient is consistent with an observed role of sphingolipids in trafficking [48], [56]. Acyl chain length is also essential for membrane dynamics since the reduction of sphingolipids containing VLCFAs increased endosome contact time and delayed membrane fusion [53]. A very long acyl chain may favor membrane curvature and thus stabilize membrane fusion intermediates during vesicle interaction.

### 1.7 Sphingolipid phosphorylation and signaling

Apart from their role as essential membrane components, sphingolipids have shown involvement in various stress responses in plants. For example phytosphingosine-1-phosphate and phosphorylated ceramides have been implicated in the plant response to chilling [20], [21] and the action of the ∆^8^-desaturase is critical in the tolerance to cold [14]. While unsaturation could still be related to membrane structural properties like fluidity to explain cold tolerance, the phosphorylated products are probably related to signaling. Ceramide phosphorylation is important for cold tolerance as reported recently by the analysis of the ceramide kinase ACD5 [21]. The role of the phosphorylated derivative of sphingosine (d18:1 ∆^4^) was also associated in ABA-dependent stomata closure and the response to drought: direct application of sphingosine-1P induced stomata closure via G protein-coupled receptor mediated pathway [17], [57]. The effect of sphingosine phosphorylation was confirmed in *SPHINGOSINE KINASE 1* mutant or overexpressing lines that respectively decrease or increase stomata sensitivity to abscisic acid [70]. However the analysis of *Arabidopsis* Δ^4^-desaturase mutant lacking both sphingosine and sphingosine-1P revealed no developmental defect (including stomata closure) suggesting most probably the involvement of another LCB [51].

Recent experiments have indicated the involvement of sphingolipids in the plant response to hypoxia. Acyl-CoA-Binding Protein 3 (ACBP3) is a membrane-anchored lipid-binding protein with a role in the modulation of hypoxia tolerance. When the gene encoding ACBP3 was disrupted or over expressed the sphingolipids containing VLCFA were subject to significant changes from the wild type. This data in combination with in vitro micro-scale thermophoresis analysis where the recombinant ACBP3 protein bound VLC acyl-CoA esters with high affinities indicated that GIPCs containing VLCFAs are essential for activation of hypoxia response and for protecting *Arabidopsis* from hypoxic stress [72], [73].

Ceramides are also implicated in a protective strategy for hypoxic tolerance in *Arabidopsis*. Experiments by [72], [73] demonstrated that hypoxia led to an elevation of ceramides and hydroxyceramides. Disruption of ceramide synthases LOH1, LOH2 and LOH3 enhanced plant sensitivity to dark submergence, but displayed more resistance to submergence under light than wild type. Levels of ceramide species containing unsaturated VLCFA declined in the LOH1, LOH2 and LOH3 mutants under dark submergence and a significant reduction of ceramides containing VLCFA in the LOH1–1 LOH3–1 double mutant was observed. The findings demonstrate that unsaturation of the VLCFA in ceramides is a protective strategy for hypoxic tolerance in *Arabidopsis* [72], [73].

Several reports describe the importance of sphingolipids in biotic response and the defense against bacteria and fungi pathogens by inducing programmed cell death (PCD). Initially the ceramide kinase ACD5 (Accelerated Cell Death) [8], and a sphingosine transfer protein ACD11 [39] were identified in screens for spontaneous PCD in absence of pathogens. The loss of ACD5 function and parallel ceramide accumulation induced ROS (reactive oxygen species) synthesis and cell death [5]. The IPC synthase gene ERH1 induced high levels of ceramides and thus spontaneous induction of cell death when mutated. It was identified as a suppressor of *RPW8* mediated resistance to powdery mildew [68]. Altogether these observations indicated that any mutation resulting in increased levels of ceramide, caused cell growth arrest and PCD in plant. As expected, ceramide phosphorylation and the transport of Cer-1P is a protective factor against PCD, since they would prevent ceramide accumulation [39]. Interestingly, mycotoxins such as AAL (isolated from *Alternaria alternata f.*sp*. lycopersici*) or FB1 inhibit ceramide synthase and induce PCD. Inhibition of ceramide synthase leads to ceramide depletion but also free LCB accumulation. Evidence suggests that free LCBs are a potential trigger signal for PCD. AAL-induced death symptoms can be suppressed by the addition of myriocin, an inhibitor of SPT [62] that reduced free LCB accumulation. Likewise, the *LCB1* mutant deficient in SPT activity was not able to accumulate free LCBs and was resistant to FB1 treatment [61]. Conversely, the application of exogenous LCBs (d18:0, t:18:0, t:18:1) induced ROS production via calcium release to induce pro-death responses ([35], [61]. It is however not clear whether a rheostat involving the ratio of LCB/LCB-P is involved in plant PCD. A mutation in the unique lyase AtDPL1 that hydrolyses LCB-P provided hypersensitivity to FB1 suggesting that mycotoxin toxicity was enhanced by both LCB-P and LCB accumulation [67]. The mutant *atdpl1* was found tolerant to the necrotrophic fungus *Botrytis cinerea* but susceptible to the hemibiotrophic bacterium *P. syringae*, suggesting a complex response of LCB-P homeostasis in biotic interactions [44].

## 2 Conclusion

In conclusion, functional analysis of sphingolipid biosynthesis demonstrated that these lipids are directly involved in many aspects of plant development and response to environment changes including biotic or abiotic stimuli. Sphingolipids are essential to basic cellular functions including membrane organization and dynamics but also to more integrated functions by their central role in the signaling for programmed cell death. Great strides have been made in the study of plant sphingolipids in the last 10 years. While the much of the biosynthesis is understood and many but not all of the genes involved have been identified and characterized there are still gaps in our knowledge. These include the regulation and control of sphingolipid synthesis and turnover as it is clear that many of these reactions are tightly controlled due to the tissue specific nature of the sphingolipid composition and the cellular distribution of sphingolipids. Little is also understood about the transport and trafficking of sphingolipids, and the precise signaling roles which are likely to be complex in nature. The specific functions of the different classes of sphingolipids or even the different moieties (LCB, acyl chain, glycans) remain future challenges for plant lipid biology.

## Transparency document

Transparency document. Image 1
